# Bacteriophage-loaded functional nanofibers for treatment of *P. aeruginosa* and *S. aureus* wound infections

**DOI:** 10.1038/s41598-023-35364-5

**Published:** 2023-05-23

**Authors:** Tobias Kielholz, Felix Rohde, Nathalie Jung, Maike Windbergs

**Affiliations:** Institute of Pharmaceutical Technology and Buchmann Institute for Molecular Life Sciences, Goethe University Frankfurt, Max-Von-Laue-Str. 9, 60438 Frankfurt am Main, Germany

**Keywords:** Antimicrobial therapy, Drug delivery

## Abstract

The increasing incidence of infected skin wounds poses a major challenge in clinical practice, especially when conventional antibiotic therapy fails. In this context, bacteriophages emerged as promising alternatives for the treatment of antibiotic-resistant bacteria. However, clinical implementation remains hampered by the lack of efficient delivery approaches to infected wound tissue. In this study, bacteriophage-loaded electrospun fiber mats were successfully developed as next-generation wound dressings for the treatment of infected wounds. We employed a coaxial electrospinning approach, creating fibers with a protective polymer shell, enveloping bacteriophages in the core while maintaining their antimicrobial activity. The novel fibers exhibited a reproducible fiber diameter range and morphology, while the mechanical fiber properties were ideal for application onto wounds. Further, immediate release kinetics for the phages were confirmed as well as the biocompatibility of the fibers with human skin cells. Antimicrobial activity was demonstrated against *Staphylococcus aureus* and *Pseudomonas aeruginosa* and the core/shell formulation maintained the bacteriophage activity for 4 weeks when stored at − 20 °C. Based on these promising characteristics, our approach holds great potential as a platform technology for the encapsulation of bioactive bacteriophages to enable the translation of phage therapy into clinical application.

## Introduction

**Figure 1:**
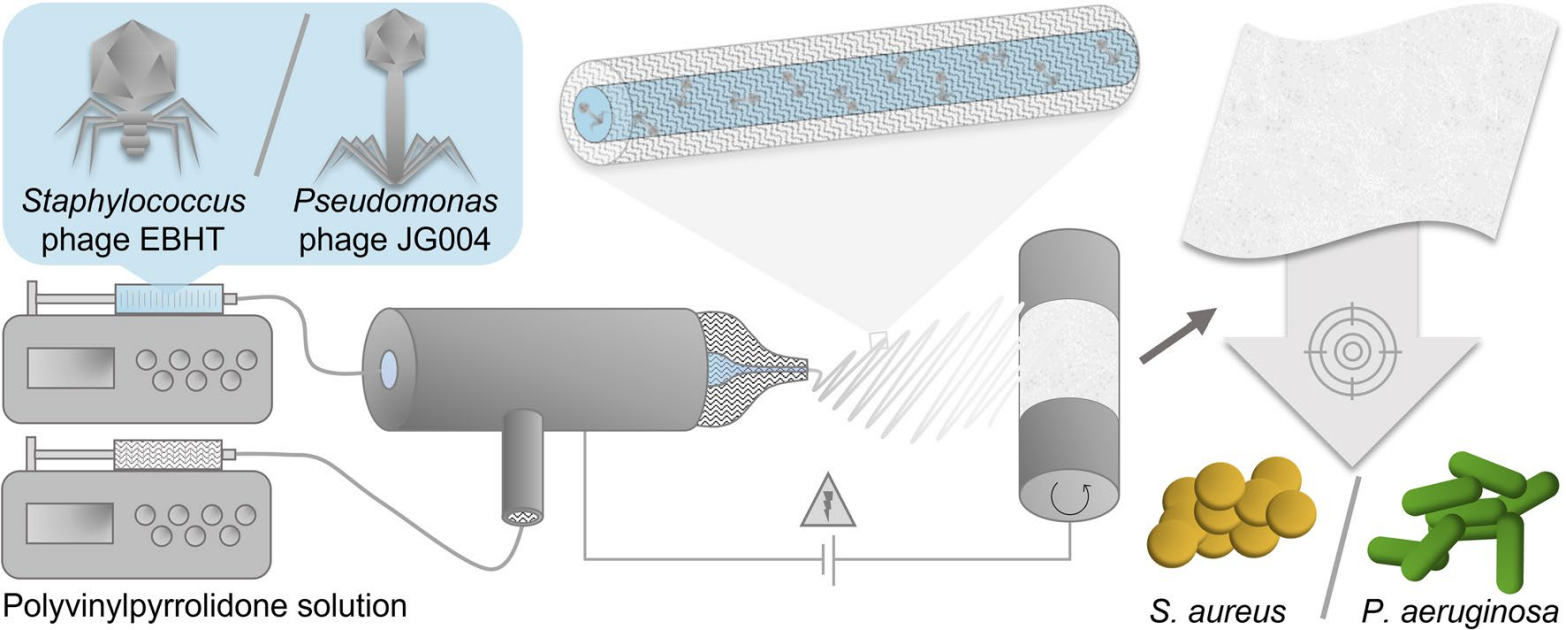
Schematic illustration of the fiber mat fabrication process employing coaxial electrospinning. The polymer solution is fed into the shell compartment of the coaxial spinneret using a syringe pump. Analogously, an aqueous dispersion of bacteriophages is pumped into the separate core compartment. After high-voltage application, electrospun fibers are deposited on a rotating drum collector, yielding a fibrous wound dressing for the treatment of infections.

In recent decades, the incidence of antibiotic-resistant infections with highly virulent ESKAPE pathogens (*Enterococcus faecium*, *Staphylococcus aureus*, *Klebsiella pneumoniae*, *Acinetobacter baumannii*, *Pseudomonas aeruginosa*, and *Enterobacter spp.*) has increased dramatically, specifically in the context of dermal wound infections^1–3^. The manifestation of secondary infections and associated complications in clinical practice make ESKAPE pathogens a leading cause of mortality worldwide, prompting the World Health Organization to encourage research on these pathogens and their targeted therapy^4,5^. The treatment of intractable antimicrobial-resistant bacterial infections emerging due to indiscriminate prescription of systemic antibiotics and the limitation of further resistance require new therapeutic strategies beyond the responsible therapeutic management of antibiotics^4,6^. Microorganisms exploit several mechanisms to protect the cell against common antibiotics and thus acquire resistance, predominantly via drug inactivation, drug efflux, or by target modification by mutation or plasmid transfer^6–8^. In this context, bacteriophages are a promising therapeutic approach. Lytic bacteriophages attach specifically to the surface of the host bacterium and inject genetic material for the replication of new virions within the bacterium. The replication of virions increases the osmotic pressure, which ultimately leads to the destruction of the cell membrane while simultaneously releasing new bacteriophages^9,10^. Although bacteriophages (phages) do not induce antimicrobial resistance as easily as conventional antibiotics, bacteria still develop surface alterations to inhibit phage entry. However, this alteration causes bacteria to reduce their motility and increase their permeability, and is therefore still considered seminal therapy^11^. It has also been shown that bacteria indirectly inhibit bacteriophage activity by stimulating the host's innate immune system^12^. However, in contrast to the extensive adverse effects of regular antibiotics, bacteriophage-based therapies have been well tolerated in clinical studies and several bacteriophages have received the GRAS (generally recognized as safe) status from the U.S. Food and Drug Administration^13,14^. For the successful delivery of bacteriophages to the local wound bed, it is necessary to enable direct contact with the infected tissue and targeted bacteria. Therefore, local delivery of bacteriophages to infected skin wounds is essential, further requiring the consideration of additional demands of topical drug delivery systems in the wound environment. It is necessary to design a carrier formulation for local delivery that optimally adapts to the irregular topography of the wound bed, but without additional mechanical stress on the tissue, to facilitate wound healing, while simultaneously preserving the biological activity of the embedded bacteriophages to ensure efficient treatment of the infection. In addition, the formulation must demonstrate good biocompatibility and it is preferable to allow for high drug loads^15^. Current treatment options comprise mainly liquid or semi-solid formulations, such as pure bacteriophage lysates or gels, which can be readily administered. Unfortunately, these formulations need to be spread throughout the infected area to achieve a homogeneous distribution, which exerts certain mechanical stress on the tissue and might impede wound healing. In contrast, wound dressings comprised of polymer fibers combine a perfect lining of the wound shape, biocompatibility, and controlled and efficient liberation of the active pharmaceutical ingredient (API) without mechanical stress upon administration. Such wound dressings can be fabricated by electrospinning, a versatile approach for the preparation of ultrathin and continuous fibers by applying an electric field to a polymer solution flux (Fig. 1)^16,17^. The technique further allows for the uniform encapsulation of APIs and has already been described in different setups for bacteriophages, such as T4 and T7 against *Escherichia coli *^18–20^. However, the current literature does not describe the rational design of fiber-based formulations for the delivery of phages for the treatment of wound infections associated with clinically relevant pathogens. As bacteriophages are only capable to infect bacteria as long as they remain biologically active, the conservation of their antibacterial activity constitutes a key factor. It has been shown, that electrospun fibers consisting of a single polymer phase are inferior to fibers with a core–shell geometry, which provide a polymer shell that protects the liquid core, thereby limiting the exposure of bacteriophages to rapid dehydration during fiber formation^20–22^. Coaxial electrospinning employs a specialized spinneret with an inner needle, surrounded by a concentric second needle, both delivering distinct phases, ultimately creating a protective polymer shell around the bacteriophage-containing core (Fig. 1)^16^. In this study, we designed bacteriophage-loaded polymer wound dressings with core–shell-structured fibers, encapsulating the *Staphylococcus* phage EBHT or the *Pseudomonas* phage JG004, to treat the highly virulent pathogens *Staphylococcus aureus (S. aureus)* and *Pseudomonas aeruginosa* (*P. aeruginosa),* respectively. The electrospun wound dressings consist of fibers with a water-soluble polymer shell surrounding the phage lysate-containing core and are intended to dissolve rapidly upon contact with wound exudate, releasing the entire bacteriophage load directly to the wound bed. A comprehensive characterization of the resulting fiber mats was performed regarding their physicochemical properties, (interior) morphology, biological compatibility, antimicrobial efficacy, and stability, revealing the formulation as a robust platform for the local delivery of phages to infected wounds.

## Results

### Fabrication and morphology of the fibers

**Figure 2:**
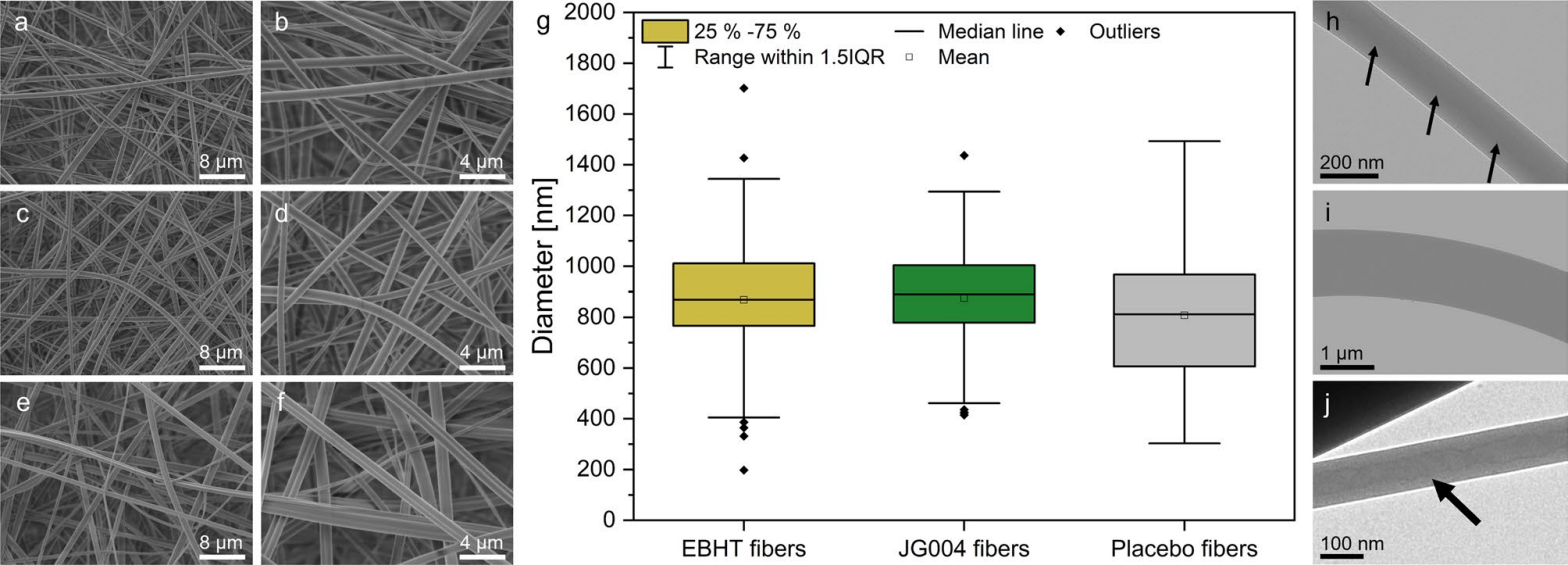
Scanning electron micrographs depicting fibers at 2500× and 5000× magnification of EBHT fibers (**a**, **b**), JG004 fibers (**c**, **d**), and placebo fibers (**e**, **f**). Box-whisker plots showing the fiber diameter distribution of bacteriophage-loaded and placebo fibers (**g**). Transmission electron micrographs of EBHT fibers (**h**, 43,000×), JG004 fibers (**i**, 9300), and placebo fibers (**j**, 9300×). Arrows indicate the core layer (**h**, **j**).

Core–shell fibers were fabricated based on the polymer polyvinylpyrrolidone (PVP) encapsulating either EBHT or JG004 phages in the form of their lysates. The shell of the fibers consists of PVP as an aqueous-soluble polymer, providing rapid and quantitative bacteriophage release upon contact with small amounts of wound exudate. Liquid bacteriophage lysates constitute the core of the fibers, with EBHT phages targeting *Staphylococcus aureus*, or JG004 phages inhibiting *Pseudomonas aeruginosa*, both representing prevalent wound pathogens. Scanning electron microscopy was used for the initial visualization of the surface morphology of fiber mats. The respective micrographs (Fig. 2a–f) revealed homogeneous, unaligned, and flawless polymer fibers with smooth surfaces, and no visual difference was found between bacteriophage-loaded and placebo fibers. Placebo fibers exhibited a broad diameter distribution ranging from 300 to 1500 nm with an average of 808 nm ± 238 nm. The fiber diameters of EBHT and JG004 samples were similarly distributed with an average of 869 nm ± 240 nm for EBHT fibers and 875 nm ± 214 nm for JG004 fibers. However, a few considerably smaller fibers were apparent, especially for the EBHT fibers (Fig. 2a, b). This group of thinner fibers was again retrieved as outliers in the corresponding box-whisker plot (Fig. 2g). Scanning electron micrographs were further acquired of the bacteriophage-loaded fiber specimens after four weeks of storage at ambient temperature (AT), 5 °C, and − 20 °C, to assess structural material alterations. Here, fibers still exhibited smooth surfaces and unaltered morphologies despite the applied storage conditions (supplementary information, Fig. S5).

As a next step for the evaluation of the resulting fiber mats and the verification of a successful coaxial electrospinning process, transmission electron micrographs were acquired to disclose the interior structure of the fiber. Figure 2h revealed that the EBHT fibers exhibited a layered architecture with a distinct shell structure around a slightly laterally shifted core. However, electrospun fibers with thicker diameters than approximately 300 nm were not translucent enough, concealing the internal structure (Fig. 2i). Only opaque fibers were visible in all the JG004 fiber samples. The placebo specimen again showed the desired core–shell fiber structure (Fig. 2j).

### Analysis of the chemical fiber composition

**Figure 3:**
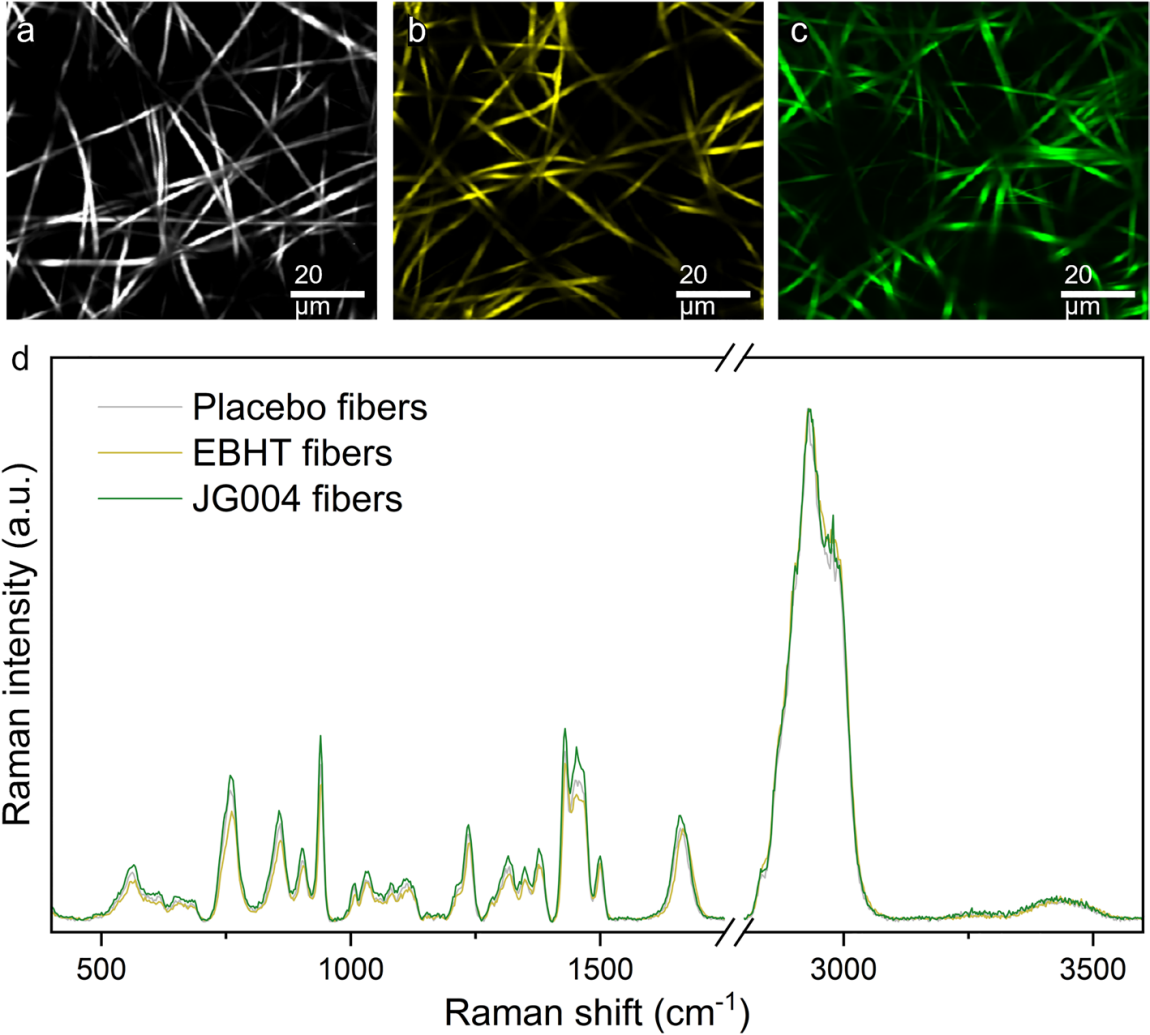
Raman false-color images of placebo fibers (**a**), EBHT fibers (**b**), JG004 fibers (**c**), and the corresponding Raman fiber spectra (**d**).

As a next step, confocal Raman microscopy analysis was performed to spatially visualize the fiber's chemical composition. False-color Raman scans (Fig. 3a–c) revealed fibers with a homogeneous chemical composition without any apparent morphological anomalies or defects. Despite minor differences in scattering intensity, the corresponding Raman spectra shared a pronounced similarity among all specimens (Fig. 3d) as well as with the polyvinylpyrrolidone (PVP) reference spectra (supplementary information, Fig. S1). Fiber spectra exhibited sharp bands at 939 cm^−1^, representing the typical PVP pyrrolidone ring breathing. Raman CH deformation peaks can be seen at 1349 cm^−1^ and 1377 cm^−1^, while peaks between 1430 and 1500 cm^−1^ correspond to CH_2_ scissor vibrations. Weak and broad OH vibration bands can be observed outside the fingerprint region in the range of 3200 cm^−1^ and 3600 cm^−1^.

**Figure 4:**
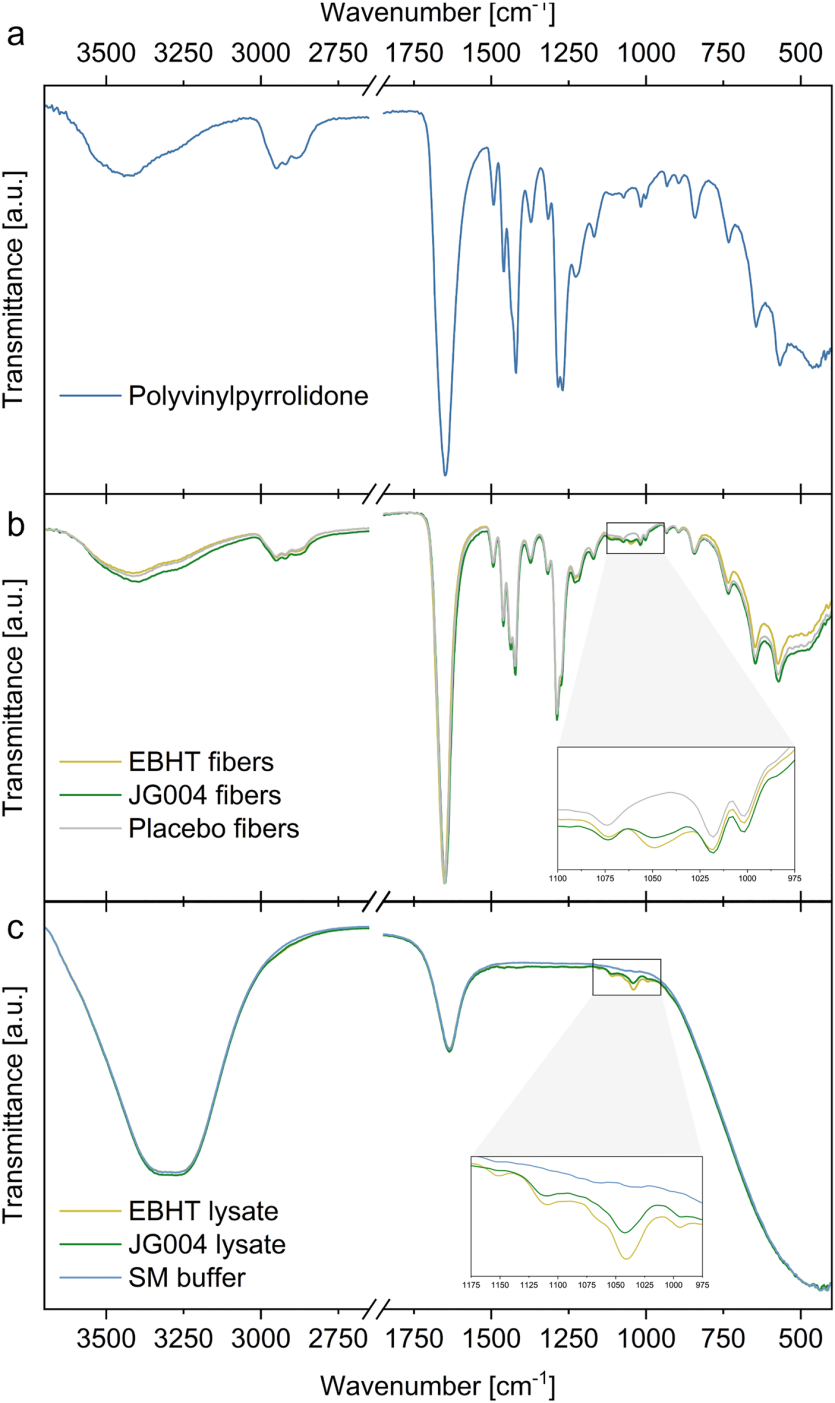
Infrared spectra of pure components (**a**, **c**) and the corresponding fiber mats (**b**).

Additionally, infrared spectra were acquired to assess the chemical composition of pure compounds of the spinning solutions and the resulting fiber mats. The reference spectra of pure PVP exhibited several CH_2_ peaks at 1461 cm^−1^, 1420 cm^−1^, 1372 cm^−1^, and 1284 cm^−1^ and were further dominated by a typical CO band at 1650 cm^−1^ (Fig. 4a). All fiber mats presented spectral patterns similar to PVP (Fig. 4b). However, the EBHT and JG004 fiber spectra represented a slight deviation from the placebo fiber spectra in the lower wavenumber region, and weak additional bands at 1049 cm^−1^ were found for bacteriophage-loaded fibers but not for the placebo formulation. This spectral feature, even though red-shifted to 1041 cm^−1^, was also apparent for the bacteriophage lysates but not for the plain storage media buffer (Fig. 4c).

### Mechanical properties of the wound dressings

**Figure 5:**
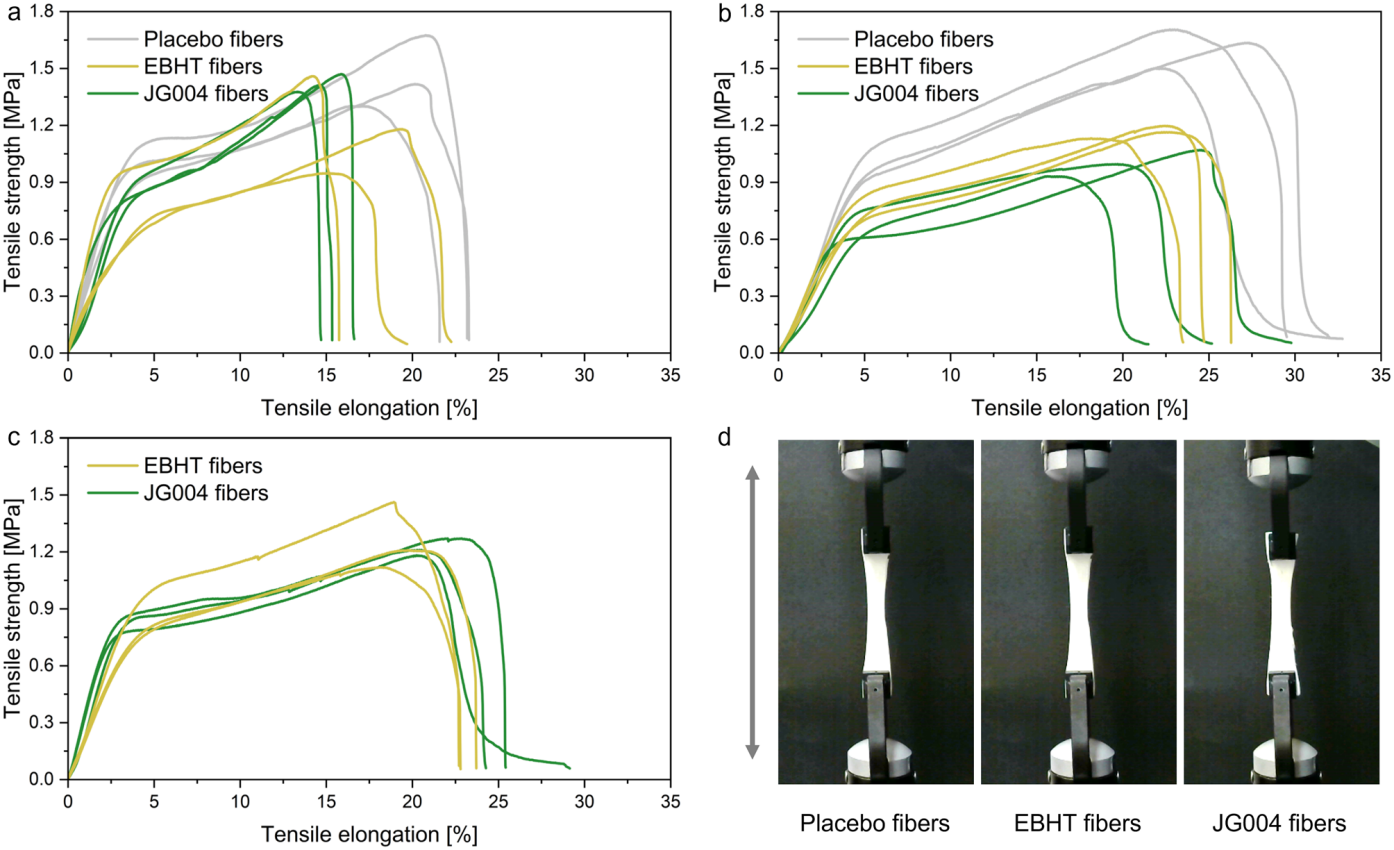
Stress–strain curves of fiber mats (n = 3) being tested in collector rotational direction (**a**), perpendicular to the collector rotation direction (**b**) and bacteriophage-loaded fiber mats after 4 weeks of storage stability testing at − 20 °C (**c**). Representative photographs of perpendicularly tested samples exhibiting necking (**d**).

The mechanical characterization of the fiber mats was determined in two orientations, along the direction of collector rotation (Fig. 5a) and perpendicular to it (Fig. 5b), as well as after 4 weeks of storage (perpendicular tested) at − 20 °C (Fig. 5c). The resulting stress–strain curves displayed no substantial differences in curve progression between replicates and between the three fiber types in the respective test directions. All tested specimens had a small elastic region (0–2.5%), followed by a large plastic deformation, resulting in a strain hardening and necking of the fiber mats (Fig. 5d), failing bulk-like. It was also found that the elongation parallel to the rotation direction was smaller (15–23%) compared to the specimens that were tested perpendicular to the rotation direction (20–32%). Overall, the calculated Young’s modulus of the fiber networks was greater in the rotational direction (JG004 41.5 ± 16.3 MPa; EBHT 36.9 ± 10.6 MPa; Placebo 37.3 ± 1.9 MPa) than perpendicular to the rotational direction (JG004 20.6 ± 3.1 MPa; EBHT 19.9 ± 2.5 MPa; Placebo 24.9 ± 0.7 MPa). Mechanical properties did not change considerably after storage of the scaffolds at − 20 °C.

### Biocompatibility testing

**Figure 6:**
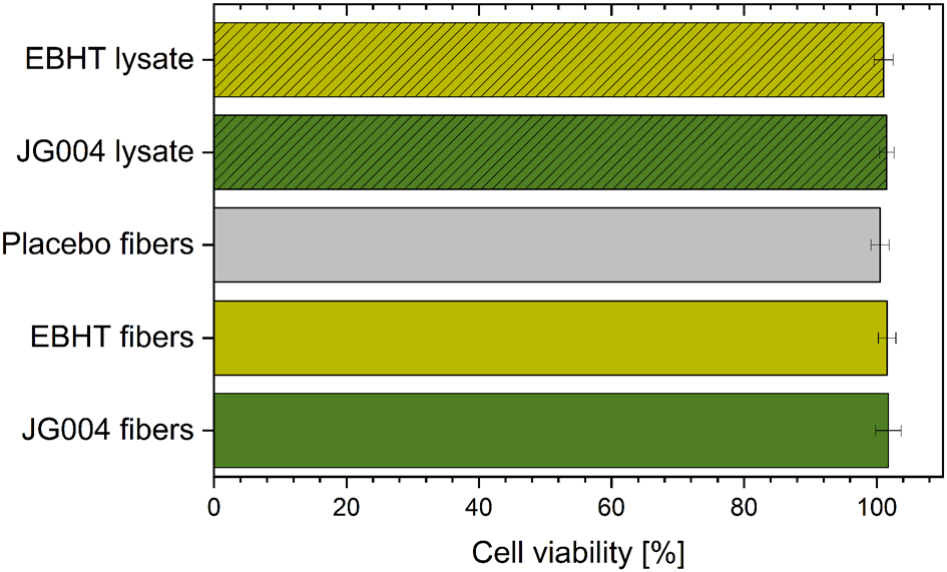
Cell viability of human keratinocytes after 24-h treatment with bacteriophage lysates, placebo, or therapeutic fiber mats.

The potential cytotoxicity of the incorporated polymers and encapsulated bacteriophage lysates was evaluated by performing a lactate dehydrogenase (LDH) assay^23^. Cell viability of human keratinocytes after 24 h of treatment was 101.1 ± 1.5% for EBHT lysate, 101.6 ± 1.1% for JG004 lysate, 100.5 ± 1.4% for placebo fibers, 101.6 ± 1.3% and 101.8 ± 1.9% for EBHT fibers and JG004 fibers, respectively (Fig. 6). No statistically significant differences were found.

### Antimicrobial performance assessment

**Figure 7:**
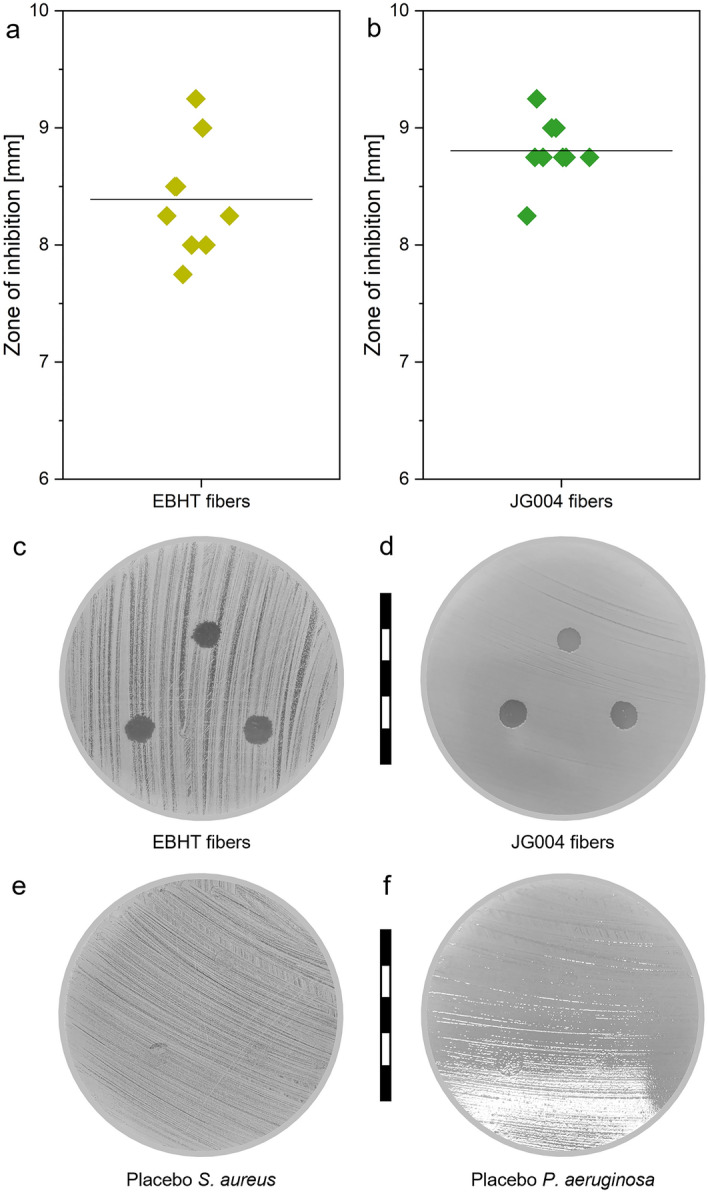
Inhibition zone diameters of EBHT (**a**) and JG004 fiber samples (**b**) against *S. aureus* and *P. aeruginosa*, respectively. Representative photographs of agar plates incubated with either bacteriophage fibers (**c**, **d**) or placebo fibers (**e**, **f**). Scale bars represent 5 cm with 1 cm intervals.

To test the antimicrobial effects of phage-loaded wound dressings, inhibition zone testing was performed using *Staphylococcus aureus (S. aureus)* and *Pseudomonas aeruginosa* (*P. aeruginosa)* as critical wound-associated pathogens targeted by EBHT and JG004 bacteriophages, respectively. Fiber samples were tested for antimicrobial activity directly after electrospinning (Fig. 7a–f). Freshly prepared EBHT fibers exhibited inhibition zones with diameters of 8.4 ± 0.5 mm (Fig. 7a), comparable to fresh JG004 fibers with 8.7 ± 0.2 mm (Fig. 7b). For both fiber types, it can be observed that samples completely dissolved upon contact with the agar surface and appeared to spread, covering a larger area than the initial 6 mm sample punch (Fig. 7c, d). Formed zones of inhibition presented a clear outline for the phage-loaded fiber samples. Testing of pure bacteriophage lysates as a control resulted in the inhibition of *S. aureus* (EBHT, 4.2 ± 0.9 mm) and *P. aeruginosa* (JG004, 3.2 ± 0.3 mm) (supplementary information, Fig. S2)*.* Additional testing of lysate-impregnated paper filter disks revealed inhibition of *P. aeruginosa* (JG004, 6.6 ± 0.2 mm) but EBHT specimens merely exhibited a halo (around the paper filter disk) with no bacterial growth (supplementary information, Fig. S2). Placebo fibers did not exhibit antimicrobial activity and samples were colonized by the respective bacteria (Fig. 7e, f).

**Figure 8:**
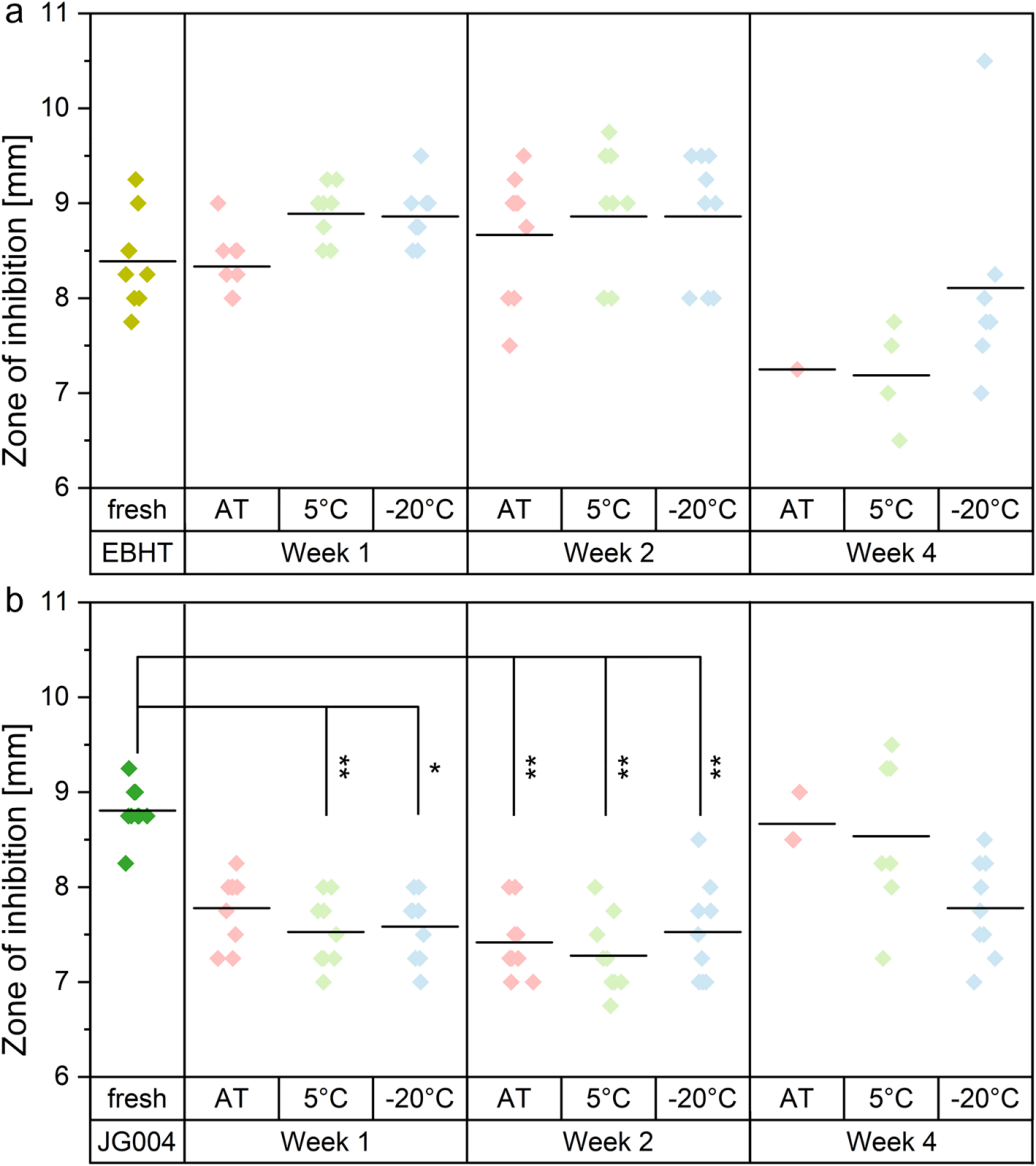
Zone of inhibition diameters of EBHT fibers (**a**) and JG004 fibers (**b**) directly after electrospinning (fresh) and after 1,2, and 4 weeks of storage at either ambient temperature (AT, red), 5 °C (green), or − 20 °C (blue).

The influence of storage time and conditions on the antimicrobial activity of bacteriophage fibers was subsequently evaluated for different storage periods (1, 2, and 4 weeks) at ambient temperature (AT), 5 °C, or − 20 °C, respectively, since the stability of proteinaceous structures such as phages is temperature sensitive (Fig. 8)^24^.

Bacteriophage fibers showed zones of inhibition after one week of storage in each condition tested. EBHT fiber mats displayed zone of inhibition diameters of 8.3 ± 0.3 mm at AT and 8.9 ± 0.3 mm for storage at both cooler conditions (Fig. 8a). *P. aeruginosa* inhibition of the JG004-loaded fibers (Fig. 8b) resulted in diameters of 7.8 ± 0.3 mm (AT) and significantly smaller diameters at 5 °C (7.5 ± 0.3 mm, *p*-value 0.0086) and − 20 °C (7.6 ± 0.3 mm, *p*-value 0.0198). After two weeks, all samples retained a pronounced inhibition with clear outlines, although the inhibition of JG004 was significantly reduced compared to fresh samples (*p*-value 0.0014 for storage at AT, 0.0001 at 5 °C, and 0.0056 at − 20 °C). After four weeks of storage, most bacteriophage-loaded fiber samples stored at AT were incapable of inhibiting their target bacteria*,* only an EBHT and three JG004 fiber samples were functional. Only four EBHT fibers (7.1 ± 0.5 mm), and seven JG004 fibers (8.5 ± 0.8 mm) were effective against the tested pathogens when stored at 5 °C. Freezing conditions at − 20 °C resulted in bacterial inhibition of seven EBHT samples (8.1 ± 1.0 mm) and all JG004 fibers retained the antibacterial potency (7.8 ± 0.5 mm).

## Discussion

Antimicrobial wound dressings need to stabilize the bioactive cargo during long-term storage and ensure its release in a quantitative and reproducible manner. In addition, fiber dressings require sufficient mechanical properties for ease of application and should not impede wound healing. Scanning electron microscopy was performed to visualize the surface morphology of the different fiber mats and to allow quantification of single fiber diameters. The electrospinning process yielded unaligned and defect-free fibers (Fig. 2a–f), which on the one hand, is important for reproducible fiber dissolution and therefore bacteriophage release. On the other hand, the random fiber orientation improves the mechanical properties of the wound dressing, allowing it to withstand tensile forces in any direction during application. Average fiber diameters ranged from 800 to 880 nm for all specimens, and samples shared a similar size distribution (Fig. 2g), indicating a consistent fabrication process. Interestingly, slightly larger fiber diameters were detected for EBHT and JG004 fibers compared to placebo fibers, suggesting a minor influence on the electrospinning process driven by the incorporation of bacteriophages in the core solution. A small proportion of fibers had substantively reduced diameters as displayed by the micrographs in Fig. 2a and c, which were subsequently identified as outliers in Fig. 2g. These thinner fibers may have resulted from cleaning the needle tip during electrospinning, which allowed smaller fibers to be formed within seconds of an interrupted process until a stable fluid jet was restored. Due to their diameter, this smaller fiber population is unlikely to harbor bacteriophages and therefore will not affect the properties of the entire fiber mat. Analysis of the internal structure of the fibers by transmission electron microscopy was important because the fiber architecture determines the efficiency of bacteriophage preservation after electrospinning^20^. Two different fiber populations were identified in the corresponding micrographs (Fig. 2a, c), but only fibers smaller than 400 nm were translucent enough to reveal the internal structure. Transmission electron micrographs (Fig. 2h, j) revealed fibers with two distinct phases, indicating the desired core–shell structure with a polymer shell and a lysate core. However, no bacteriophages were found in the core compartment of such small EBHT fibers (Fig. 2h), most likely due to steric hindrance, as bacteriophages have a size slightly smaller than the fiber diameter itself (smaller outliers, Fig. 2g). Micrographs of the placebo fibers (Fig. 2j) also exhibited two distinct phases, with no significant difference from EBHT fibers that would be indicative of dissolution of the polymer shell. Nonetheless, most samples contained fibers larger than 400 nm (Fig. 2h), as shown in the scanning electron micrographs (Fig. 2a–f), which could not be examined because even high doses of radiation did not result in transparency. The produced homogeneous and defect-free fibers with similar fiber diameter distributions represented the basis for the subsequent analysis steps.

The implemented spectroscopic analytical approaches allowed for the chemical identification and visualization of the electrospun fiber mats. Spatially resolved chemical imaging via confocal Raman microscopy detected the respective PVP Raman peaks in accordance with the literature and revealed an even chemical distribution in all analyzed fiber types (Fig. 3)^25,26^. Corresponding Raman spectra of bacteriophage-loaded fibers did not reveal a substantial difference in the form of additional Raman peaks compared to those of placebo fibers, indicating intact PVP polymer shell structures shielding the biological cargo in the core. However, Raman spectra of fibers showed no evidence of biological traces, such as specific bands indicative of bacteriophages. Attenuated total reflection infrared (ATR-IR) spectroscopy was performed as an additional chemical analysis, and corresponding spectra confirmed the presence of unaltered polymer fibers with characteristic PVP bands as depicted by the PVP reference spectra (Fig. 4)^27^. Furthermore, a distinct peak between 1040 and 1050 cm^−1^ was found to be exclusively present in samples loaded with bacteriophages, confirming the successful bacteriophage encapsulation. This particular peak could be attributable to C–O vibrations of carbohydrates in the nucleic backbone^28–30^. Of the two implemented spectroscopic approaches, only ATR-IR spectra provided valid proof of bacteriophages in the electrospun fiber mats. Probably due to pressure applied during the ATR-IR spectroscopy sample fixation and measurement, the integrity of core–shell fibers was impaired and core fluid was exposed, resulting in the detection of leaked core fluid. Confocal Raman spectroscopy, in contrast, as a non-destructive and contact-free analysis approach, was not able to detect Raman peaks originating from the core, as they are likely to be covered by strong polymer signals from the surrounding shell.

After the chemical analysis of electrospun fiber mats, the mechanical properties of fiber networks were evaluated. The mechanical performance of electrospun fiber mats is affected by several factors, such as fiber diameter, architecture of the fiber network, or polymer selection^31,32^. Also, collector rotation speeds may result in oriented fiber deposition, thus posing another critical factor. Therefore, uniaxial tensile testing was conducted parallel and perpendicular to the rotation direction of the collector to determine mechanical properties in both directions and to detect anisotropy caused by fiber alignment. The resulting stress–strain curves display similar mechanical properties and failure characteristics for each specimen tested, confirming the isotropic (direction-independent) properties of the scaffolds. The increase in the determined Young’s (elastic) modulus and the decrease in elongation for the tensile-tested fiber mats in the spinning direction occurs due to the low degree of fiber alignment at a rotational speed of 2 m/s. As a result, fibers parallel to the rotational direction resist a higher tensile stress load and are not reorganized within the fiber mesh, ultimately leading to increased stiffness and shortened elongation^33^. The values obtained for Young’s moduli are similar to the elastic moduli of human skin (approximately 30 MPa), thus making the scaffold as elastic as human skin (Fig. 5)^34^. Considering these results, the random fiber orientation results in favorable direction-independent mechanical properties that ease wound dressing handling and administration. In addition, the mechanical properties of the scaffolds were not altered by storage at − 20 °C, confirming that the freezing and thawing process does not alter the structural stability of the polymeric shell (see also supplementary information Fig. S5), thus allowing adequate storage of bacteriophage-loaded wound dressings without detrimental loss of mechanical performance.

In order to exclude any potential cytotoxic effects after the application of the electrospun wound dressing to injured and infected human skin, human keratinocyte viability was quantified *in vitro* by conducting an LDH cell viability assay^23^. The incubation period of 24 h was selected to match the estimated application interval of the wound dressing and the use of a cellular monolayer of keratinocytes can be considered a reliable and sensitive test method to determine potential cytotoxic effects. However, none of the applied samples increased the concentration of LDH in the cell culture medium in comparison to the untreated control, attesting high cellular membrane integrity and therefore viable cells that do not undergo necrosis or apoptosis. Neither the polymer PVP that encapsulates the bacteriophages nor the pure phage lysate induced cellular damage after application, which is consistent with the literature (Fig. 6.)^35,36^. Considering these results, the electrospun wound dressings exhibit high biocompatibility and are unlikely to evoke any adverse effects when applied to human skin.

Antimicrobial performance studies were conducted to validate proper bacteriophage encapsulation and conservation of their biologically active form. Fiber mats dissolved upon contact with small amounts of adherent fluid on the agar plate, thereby releasing the entire encapsulated bacteriophages. This immediate dissolution of wound dressings on damp agar plates attests to the intended instant dissolution behavior in the wound environment and the aspired rapid delivery of bacteriophages to the infected area. Core–shell fibers were found to maintain the activity of EBHT and JG004 by displaying a successful inhibition against *S. aureus* and *P. aeruginosa* (Fig. 7c, d). The average inhibition zone diameter of both fiber types was considerably larger than the initial diameter of the applied fiber mats (Fig. 7a, b). However, this observation was not related to the diffusion of bacteriophages, but rather to the spreading of dissolved bacteriophage-containing PVP fibers on the agar surface, as seen with the placebo fibers, which behaved analogously (Fig. 7e, f). This explanation was further underlined by the result of the bacteriophage lysate control. Here, the EBHT lysate showed zones of inhibition limited to the diameter of the applied droplet (see also supplementary information Figs. S2 and S3), emphasizing that bacteriophages do not diffuse through solid agar media like regular small molecules and peptide antibiotics^37^. However, the diameters of the JG004 lysate droplets were negligibly smaller compared to the corresponding zone of inhibition diameter, likely due to a wetter agar surface during application. Lysate-impregnated paper filter disks were chosen as a second control to allow for the application of bacteriophage lysate with an identical area to the electrospun fibers. These specimens revealed smaller zones of inhibition compared to the bacteriophage-loaded fiber mats, clearly underlining the superior performance of the electrospun wound dressings. The improved activity of fiber mats is independent of the incorporated polymer in the shell of each fiber (as placebo fiber mats showed no antimicrobial activity) and can be explained by the protective and readily soluble polymer fibers that disperse the encapsulated bacteriophages upon dissolution.

Core–shell polymer fibers maintained antimicrobial activity for multiple weeks for each tested storage condition, but after four weeks of storage at ambient temperature, almost all EBHT and JG004 fibers lost their antimicrobial activity, which is consistent with the literature (Fig. 8)^20,38^. Here, the specimens were not completely ineffective, as various clear plaques were still apparent, but bacterial colonies were present in the area of the applied fiber mats, thus no complete inhibition could be stated (supplementary information, Fig. S4). Lower storage temperatures (5 °C and − 20 °C) led to the preservation of the bacteriophage activity with superior performance for fibers stored at − 20 °C. Interindividual variability (EBHT, JG004) in the number of effective fiber samples has been reported to be related to species-dependent stability versus pH and osmolality^39^. However, the zone of inhibition against *S. aureus* was less pronounced after one and two weeks of storage, in contrast to the preceding stability assessment, indicating a possible influence of the long-term storage on their antimicrobial activity. On the one hand, the area where the fiber mats were applied to the agar was free of bacterial colonies (in contrast to samples stored at ambient temperature), therefore the decrease in antimicrobial activity is most likely due to a reduced ability of the polymer fibers to spread upon dissolution and is not associated with a degradation of bacteriophages within the fiber formulation. On the other hand, scanning electron micrographs revealed flawless fiber morphologies even after 4 weeks of storage (supplementary information Fig. S5). Overall, the coaxial electrospinning approach resulted in effective antimicrobial fibers with sustained inhibition of *S.* *aureus* and *P.* *aeruginosa* over four weeks, when stored appropriately at − 20 °C.

## Conclusion

In the present study, we successfully encapsulated two clinically relevant bacteriophages in electrospun wound dressings for the treatment of *S. aureus* and *P. aeruginosa* infected skin wounds. By coaxial electrospinning, core–shell fibers were designed with bacteriophage lysates as the aqueous core surrounded by a protective PVP polymer shell. The encapsulated bacteriophages retained their activity, thus providing a pronounced antimicrobial efficacy in vitro. Fiber mats further exhibited superior biocompatibility and good mechanical properties, necessary for handling in clinical practice. The electrospun fibers developed herein represent a versatile formulation for functional bacteriophage-loaded wound dressings, allowing for customization with respect to the integration of stabilizing agents, the combination of different phages, or additional synergistic APIs for a tailored therapy of infected wounds. Thereby, the here described rational design of an adaptable fiber-based platform technology offers high potential for translation of phage therapy into clinical practice.

## Methods

### Materials

*Staphylococcus* Phage EBHT (DSM 26856), *Pseudomonas* Phage JG004 (DSM 19871), *Pseudomonas* *aeruginosa* (Schroeter 1872) Migula 1900 (DSM 19880), and *Staphylococcus aureus* Rosenbach 1884 (DSM 104437) were kindly provided by Dr. Christine Rohde from the Leibniz Institute DSMZ—German Collection of Microorganisms and Cell Cultures GmbH (Braunschweig, Germany). Sodium chloride, magnesium sulfate, tris(hydroxymethyl)aminomethane (Tris), trypticase soy broth, ethanol, Dulbecco’s phosphate-buffered saline (PBS), and hydrochloric acid were purchased from VWR International GmbH (Darmstadt, Germany). Polyvinylpyrrolidone (PVP, mass average molar mass approximately 1,300,000 g/mol) and bacteriological agar were obtained from Sigma-Aldrich (St. Louis, USA). LB agar acc. to Lennox was purchased from Th. Geyer (Renningen, Germany). A PureLab Flex 2 purification system (Veolia Water Technologies Deutschland GmbH, Celle, Germany) was utilized to produce deionized water. The Cytotoxicity Detection Kit (Roche Diagnostics, Mannheim, Germany) was purchased from Sigma Aldrich (Darmstadt, Germany). Immortalized human keratinocytes (HaCaT) were kindly provided by Prof. Dr. Fusenig (German Cancer Research Center, Heidelberg, Germany)^40^. High-glucose Dulbecco’s modified Eagle Medium (DMEM), trypsin–EDTA were purchased from Gibco™ Life Technologies, ThermoFisher (Darmstadt, Germany). Fetal calf serum (FCS) was obtained from Sigma-Aldrich (Darmstadt, Germany).

### Preparation of electrospinning solutions

For the electrospinning formulations, polyvinylpyrrolidone (PVP, 14%, w/v) was dissolved in ethanol and vigorously stirred at ambient temperature overnight. Shortly before use, the polymer solution was vortexed, degassed, and filled into a 10 mL plastic syringe from B. Braun SE (Melsungen, Germany). Bacteriophage lysates were used as provided (EBHT) or diluted in a ratio of 1:2 in storage media buffer (JG004) and subsequently transferred into a 1 mL syringe (Hamilton, Reno, USA). Storage media buffer was prepared by dissolving 5.8 g of sodium chloride, 0.96 g of magnesium sulfate, and 6 g of Tris in 1 L deionized water, and the pH was adjusted to 7.5 using 0.1 M hydrochloric acid. Storage media buffer is a general-purpose phage buffer recommended by the Leibniz Institute DSMZ—German Collection of Microorganisms and Cell Cultures GmbH (Braunschweig, Germany) and was also used to prepare placebo fibers.

### Electrospinning

A stainless-steel coaxial spinneret with two concentric nozzles (inner needle gauge 22, outer needle gauge 18; ramé-hart instrument co., Succasunna, USA) was used to produce fibers with a polymer shell and an aqueous core consisting of bacteriophage lysate or lysate diluted with storage media buffer. Prior to electrospinning, the coaxial spinneret was mounted on the 1 mL syringe containing the core solution, and the syringe with the 10 mL polymer shell solution was connected to the spinneret using a polypropylene tubing and Luer-Lock connectors. Electrospinning was carried out with a flow rate of 2.5 mL/h for the polymeric shell solution and 150 µL/h for the core solution. Voltages in the range of 16–20 kV and 18–22 kV were applied during the preparation of EBHT and JG004 fibers, respectively. The applied voltage was increased over the course of the electrospinning process to compensate for the electrical insulation caused by fiber deposition on the collector. In the manuscript, the resulting fiber mats are abbreviated as EBHT fibers, and JG004 fibers, respectively, based on the incorporated bacteriophages. Placebo fiber mats were fabricated analogously. Fibers were deposited at 26–44% relative humidity and 22–27 °C on a metal drum collector (50 mm diameter, 150 mm width) rotating at 2 m/s at a distance of 22 cm from the needle tip for a total of 60 min.

### Scanning electron microscopy

For fiber morphology evaluation and subsequent fiber diameter determination, scanning electron micrographs were recorded using a ZEISS EVO 10 electron microscope (Carl Zeiss Microscopy GmbH, Jena, Germany) at 3 kV acceleration voltage and a probe current of 20 pA. Specimens were sputter-coated with palladium/gold for five minutes (SC7620 Mini Sputter Coater, Quorum Technologies, Lewes, UK). Fiji software^41^ was utilized for fiber diameter analysis evaluating the diameter of 100 randomly selected fibers.

### Transmission electron microscopy

To verify a successful coaxial electrospinning process, the internal structure of the electrospun fibers was resolved by transmission electron microscopy (TEM) using a Tecnai Spirit BioTwin (FEI Company, Oregon, USA) equipped with a Gatan bottom-mount US4000 CCD camera (4 k × 4 k, Gatan, Inc., Pleasanton, USA). Fibers were deposited directly onto a carbon-coated copper grid (300 mesh, Plano GmbH, Wetzlar, Germany) for 60 s prior to imaging at 120 kV (LaB_6_ cathode). Micrographs were processed using Fiji software^41^.

### Confocal Raman microscopy

Chemically selective imaging was performed using a confocal Raman microscope (alpha 300R+, WITec GmbH, Ulm, Germany), equipped with a 50× objective (NA 0.8, Epiplan Neofluar obtained from, Zeiss, Germany), a 532 nm diode laser (38.6 mW power before the objective), and a confocal pinhole, rejecting out-of-focus signals. Raman spectra were recorded in the spectral range of 400–3700 cm^−1^ and a resolution of 4 cm^−1^. Raman imaging was conducted for sections of 100 × 100 µm size with 0.1 s acquisition time per data point and a resolution of 0.33 µm. WITec´s ProjectFour software (WITec GmbH, Ulm, Germany) was used to apply cosmic ray removal and background subtraction to the spectral data sets followed by supervised k-means clustering and basis analysis yielding false-color Raman maps.

### Attenuated total reflection infrared spectroscopy

Attenuated total reflection infrared (ATR-IR) spectroscopy allowed for the qualitative composition analysis of the fiber mats. An ALPHA II Platinum spectrometer (Bruker Corporation, Billerica, USA) was used, recording 24 scans per data point at multiple spots across each sample. Spectra have a resolution of 4 cm^1^, were recorded in a shift range of 4.000–400 cm^−1^(OPUS software version 8.1.29, Bruker Corporation, Billerica, USA), and subsequently averaged and normalized (0,100) using Origin Pro 2022 (OriginLab, Northampton, USA).

### Tensile testing

The fiber mat’s mechanical properties were assessed with an Instron 3950 tensile tester (Instron, Norwood, USA) equipped with a 50 N load cell. Fiber mats were cut into rectangular sample strips (55 × 10 mm size) and a thickness measurement gauge (MiniTest 735, ElektroPhysik Dr. Steingroever GmbH & Co. KG, Cologne, Germany) was used to determine the corresponding sample thickness. Fiber specimens were mounted in rubber grip sample holders and consecutively stretched at 10 mm/min. Tensile stress, tensile strain, and Young’s modulus were calculated with Bluehill^®^ Universal software (Version 4.11, Instron, Norwood, USA). Three technical replicates were used to determine the mechanical properties.

### Cytotoxicity testing

Human keratinocytes were cultivated in DMEM supplemented with 10% fetal calf serum at 37 °C, 5% CO_2,_ and 80% humidity. The medium was refreshed every 2–3 days. Determination of cell viability was conducted using a lactate dehydrogenase (LDH) assay^23^ on HaCaTs seeded with a density of 4 × 10^4^ in 48 well plates (Greiner Bio-One, Kremsmünster, Österreich) and grown for 48 h until reaching 90% confluence. Circular samples of electrospun fiber mats (10 mm) were disinfected for 60 min using UV-C radiation. EBHT and JG004 fibers were used as test samples, placebo fibers and bacteriophage lysates of EBHT (0.61 µL) and JG004 (0.57 µL) were used as controls. The samples and controls were subsequently added to the HaCaT cell monolayers cultivated in 500 µL cell culture medium and incubated for 24 h (n = 6). To quantify the cell viability, a positive control (untreated cell monolayer) was incubated with an untreated cell culture medium and a negative control (treated with 2% Triton X-100 in DMEM/FCS) were tested. After 24 h, 400 µL of the supernatant was withdrawn and centrifuged at 250 rpm for 10 min. Subsequently, 100 µL were transferred in a 96-well plate (Greiner Bio-One, Kremsmünster, Österreich) and the assay reagents were added. The optical density (OD) was measured at 492 nm (Tecan Spark., Tecan, Crailsheim, Germany). The cell viability of HaCaTs was quantified according to the manufacturer’s protocol (see Eq. 1).

1 $$Cell \,\,viability \left[ \% \right] = \left( {1 - \left( {\frac{{OD_{EXP} - OD_{{CTRL^{ + } }} }}{{OD_{{CTRL^{ - } }} - OD_{{CTRL^{ + } }} }}} \right)} \right)*100\%$$

Cell viability was assessed in three biological replicates.

### Antibiotic susceptibility testing

Overnight cultures of *Staphylococcus aureus* (*S. aureus*) DSM-104437 and *Pseudomonas aeruginosa* (*P. aeruginosa*) DSM-19880 (Leibniz Institute DSMZ—German Collection of Microorganisms and Cell Cultures GmbH, Braunschweig, Germany) were cultivated at 37 °C on agar dishes (Difco™ Nutrient Agar, BD, New Jersey, USA) to generate bacterial suspensions with an optical density of 0.5 McFarland in PBS (Biowest, Nuaillé, France). The bacterial suspensions were then spread on Petri dishes with trypticase soy broth agar (30 g/L trypticase soy broth and 15 g/L agar, pH 7.3; used for *S. aureus* and EBHT samples) or Luria Bertani agar (35 g/L LB agar according to Lennox supplemented with 5 g/L agar and 5 g/L sodium chloride, pH 7.0; used for *P. aeruginosa* and JG004 samples) as recommended culture media for host bacteria. Subsequently, circular 6 mm punches of the respective fiber mats, as well as lysate droplets and 6 mm filter paper discs, impregnated with bacteriophage lysate in the equivalent dose of bacteriophage lysate used in the fiber mat samples (0.16 µL EBHT, 0.2 µL JG004 per 6 mm punch), were administered. After 24 h of incubation at 37 °C, a caliper was used to measure the zone of inhibition. Antibiotic susceptibility was determined using biological triplicates with three technical replicates each.

### Stability studies

Fiber mats were stored under different conditions: at ambient temperature, 5 °C, and − 20 °C in containers together with a desiccant. Antimicrobial performance was assessed after 1, 2, and 4 weeks. Mechanical properties were evaluated after 4 weeks.

### Statistical analysis

Statistical analysis was applied for the eucaryotic cytotoxicity and antimicrobial susceptibility tests. The presence of statistically significant differences (alpha level 0.05) between groups was determined with Kruskal–Wallis tests, and two-tailed Dunn’s tests were employed (Origin Pro 2022, OriginLab, Northampton, USA) to generate the corresponding P-values as samples are independent and calculations do not depend on the normality of populations. *p* values < 0.05 were considered statistically significant (*p < 0.05, **p < 0.01).

## Supplementary Information

Supplementary Figures.

## Data Availability

The datasets generated during and/or analyzed during the current study are available from the corresponding author on reasonable request.

## Abbreviations

API: Active pharmaceutical ingredient
AT: Ambient temperature
ATR-IR: Attenuated total reflection infrared spectroscopy
CH_1/2/3_: Carbon-hydrogen bonds
C-O: Carbon–oxygen bond
FCS: Fetal calf serum
GRAS: Generally recognized as safe
HaCaT: Human keratinocyte
LDH: Lactate dehydrogenase
OD: Optical density
OH: Oxygen-hydrogen bond
PBS: Phosphate-buffered saline
PVP: Polyvinylpyrrolidone
