# Supplementary material for: Bacteriophage-loaded functional nanofibers for treatment of P. aeruginosa and S. aureus wound infections
Source: Sci Rep. 2023 May 23;13:8330. doi: 10.1038/s41598-023-35364-5 (PMC10205809; doi:10.1038/s41598-023-35364-5)
Supplement: Supplementary file 1 — Supplementary Figures. [file 41598_2023_35364_MOESM1_ESM.docx]

**Supplementary information – Fig. S1: Polyvinylpyrrolidone Raman spectra
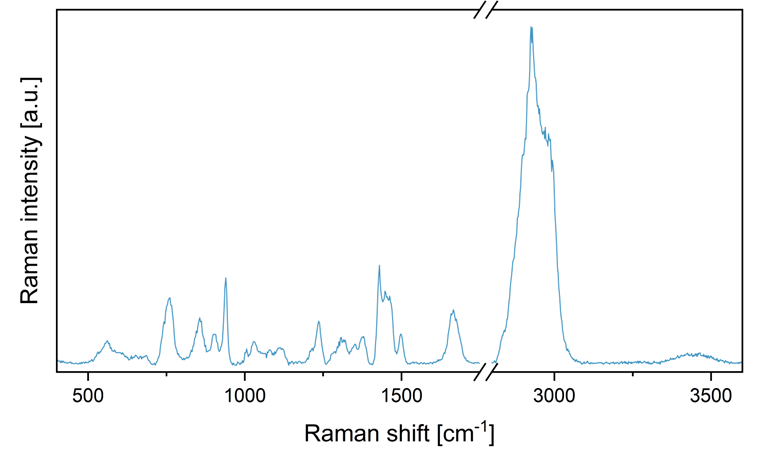
**

Figure S1: Raman spectra of the polyvinylpyrrolidone reference substance.

**Supplementary information – Fig. S2: Antimicrobial susceptibility testing of control samples**


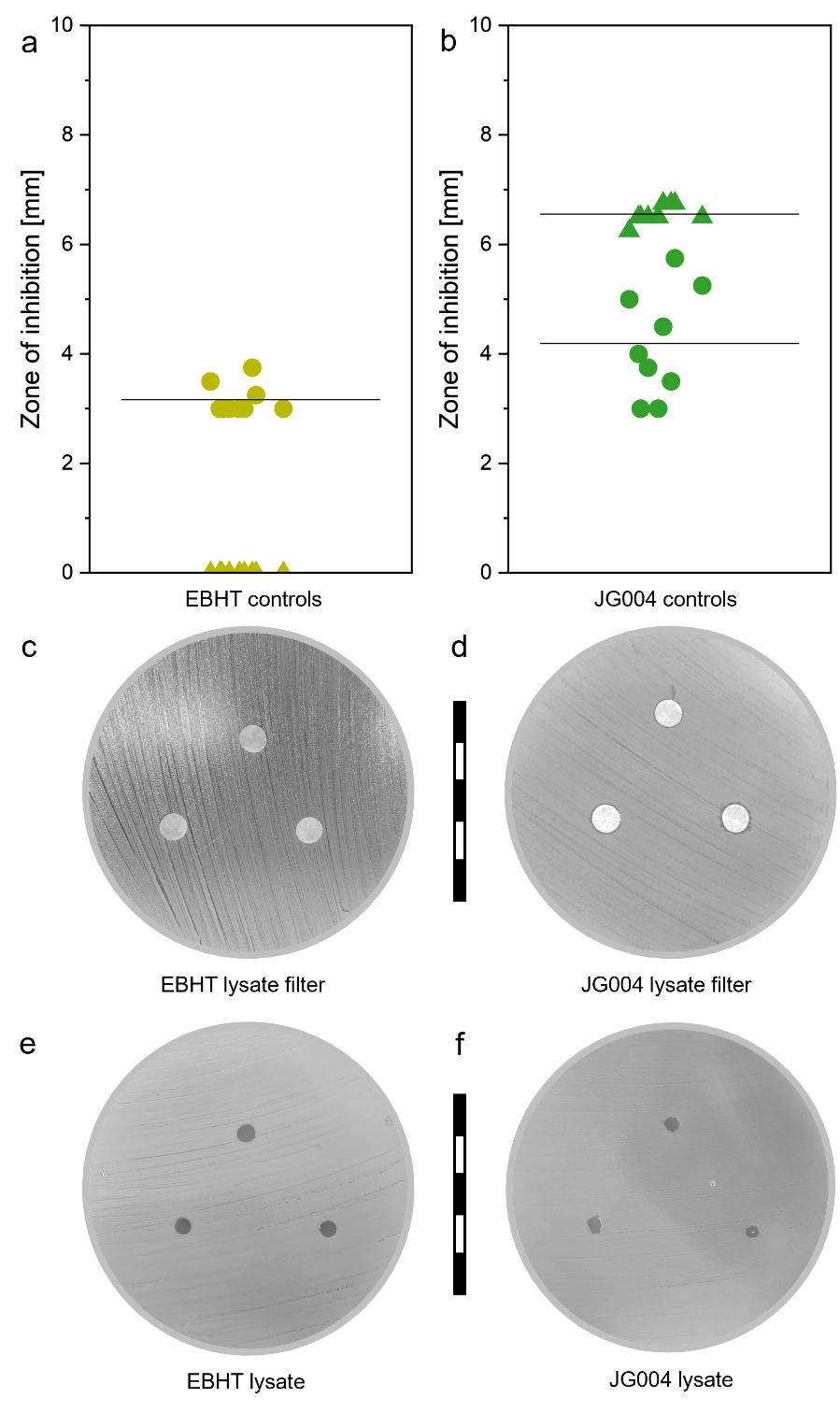


Figure S2: Zone of inhibition diameters of bacteriophage control samples of EBHT (a) and JG004 (b) with lysate impregnated filter disks (triangles) and pure lysate (circles). Photographs of lysate impregnated control samples (c,d), and pure lysate control (e,f).

**Supplementary information – Fig. S3: Wettability assessment for determination of lysate droplet diameters**


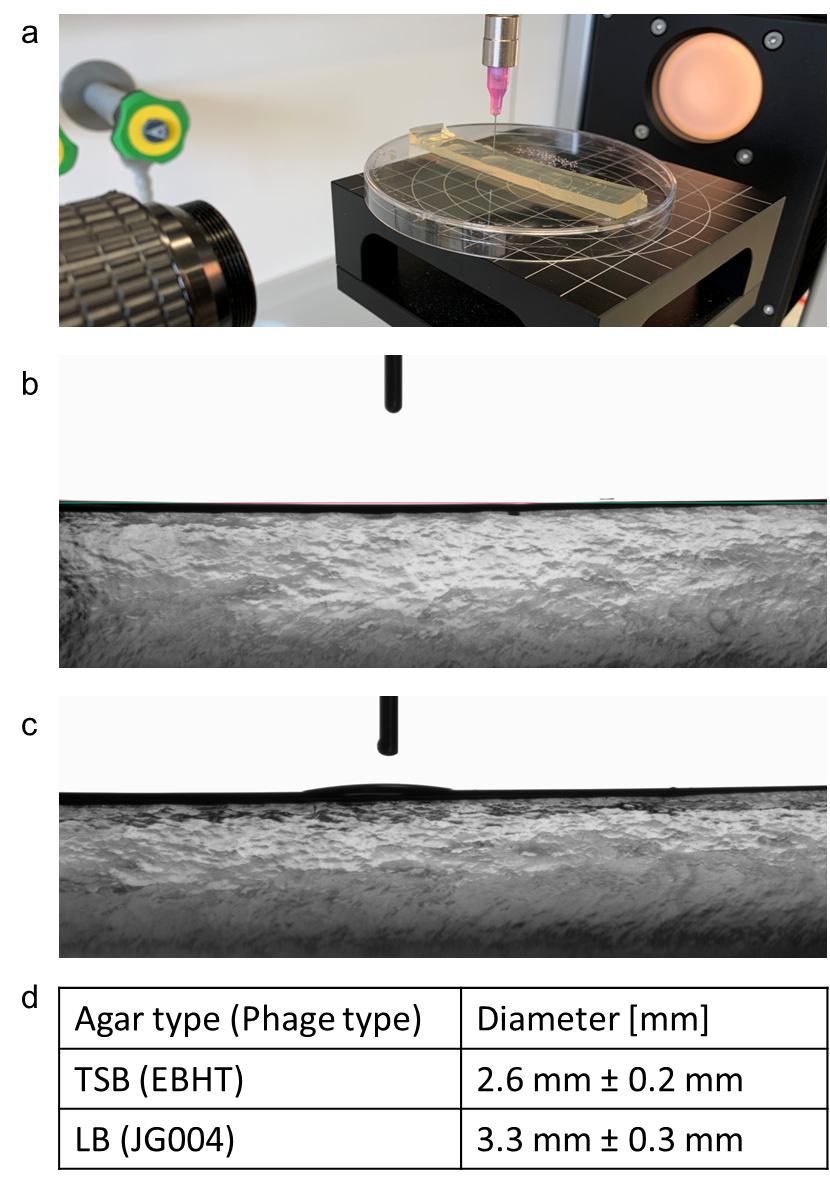


Figure S3: Experimental setup of the wettability assessment for the determination of lysate control droplet diameters (a). A rectangular slice of Luria Bertani agar (LB) or trypticase soy broth agar (TSB) was placed on a Petri dish lid and inoculated with phosphate-buffered saline, mimicking the bacterial suspension inoculation (b). Afterward, 0.16 µL (EBHT lysate samples and TSB agar) or 0.2 µL (for JG004 lysate samples and LB agar) storage media buffer was applied (c), and the resulting diameter was calculated with the SCA20 software (V 5.0.37, DataPhysics Instruments, Germany). Respective results of the diameter evaluation (d).

**Supplementary information – Fig. S4: Inhibition for fibers stored at ambient temperature**


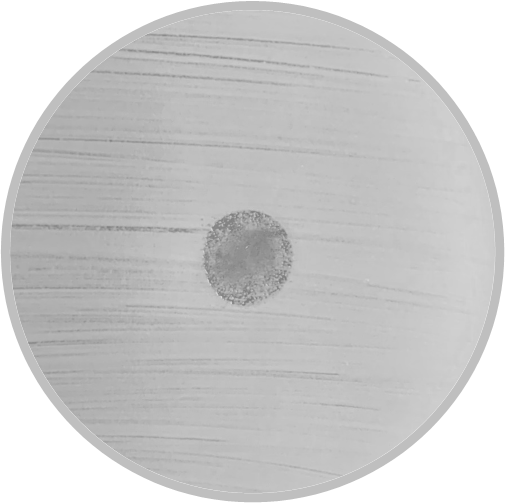


Figure S4: Photograph depicting the result of a zone of inhibition test with EBHT fibers after four weeks of storage at ambient temperature. specimens were not completely ineffective as various clear plaques are still apparent, but bacterial colonies are present in between.

**Supplementary information – Fig. S5: Electrospun fibers after storage**

**
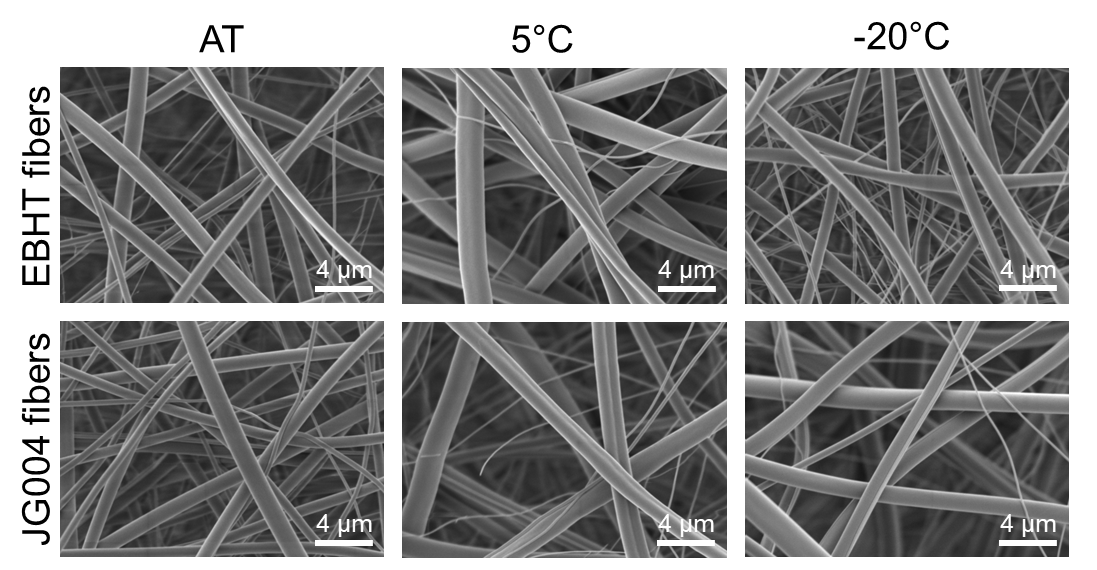
**

Figure S5: Scanning electron micrographs (5000x magnification) of bacteriophage-loaded fibers after 4 weeks of storage at different conditions: ambient temperature (AT), chilled (5°C), and frozen (-20°C).
